# Family-centred, therapeutic camps for Aboriginal children with complex neurodevelopmental needs and early life trauma in remote Australia: a qualitative study of the Marurra-U family camp

**DOI:** 10.1016/j.lanwpc.2026.101974

**Published:** 2026-09-10

**Authors:** Anita Pickard, Thomas Stubbs, Jadnah Davies, Sharanya Napier-Raman, Emily Carter, Sue Thomas, Vondella Berringal, Zarema Johnson, Jeanette Pindan, Clare Hickey, Rosemary Evans, Kim Casburn, Tuguy Esgin, Elizabeth J. Elliott, Alexandra Martiniuk

**Affiliations:** a Faculty of Medicine and Health, School of Public Health, The University of Sydney, Sydney, New South Wales, Australia; b Marninwarntikura Women's Resource Centre, Marulu Team, Fitzroy Crossing, Western Australia, Australia; c Royal Far West, Manly, New South Wales, Australia; d Dean Indigenous Faculty of Business and Law, Curtin University, Western Australia, Australia; e Centre for Social Impact, University of Western Australia, Business School, Perth, Western Australia, Australia; f The University of Sydney, Faculty of Medicine and Health, Specialty of Child and Adolescent Health, Sydney, New South Wales, Australia; g Kids Research, Sydney Children's Hospitals Network, Westmead, New South Wales, Australia; h Dalla Lana School of Public Health, The University of Toronto, Toronto, Ontario, Canada; i International Center for Future Health Systems, University of New South Wales, Sydney, New South Wales, Australia

**Keywords:** Aboriginal and Torres Strait Islander, Family-centred care, Allied health, Neurodevelopmental disorders, FASD, ELT, Adverse childhood experiences (ACEs), Therapeutic recreation camp, Parent-child, Two-way teams

## Abstract

**Background:**

Aboriginal Community Controlled Organisations in the Fitzroy Valley are internationally recognised for their leadership in supporting children with foetal alcohol spectrum disorder and early life trauma, and their families. This study investigates the design and parent-reported impact of the Marurra-U family camp, a family-centred, therapeutic, recreational camp for Aboriginal children with complex neurodevelopmental or social-emotional needs.

**Methods:**

An Aboriginal Participatory Action Approach was used. Nine yarning sessions with 13 Aboriginal parent/carers who attended the Marurra-U family camp in 2024 or 2025 were conducted. Researchers also conducted ethnographic observations whilst participating in the camps. Data were analysed using reflexive thematic analysis.

**Findings:**

Four themes were constructed: 1) The unique characteristics and design of the Marurra-U family camp, 2) Children actively engaged in camp activities, leading to improved individual, family, and social wellbeing, 3) Parents were motivated and empowered to support their children and communities to thrive, 4) Two-way teams were effective in delivering child and family support.

**Interpretation:**

Parents reported that the camps supported emotion regulation, social skills, confidence, and behaviour in children, and empowered, built knowledge, and skills in parents to support their children. This demonstrated that family-centred, therapeutic, recreational camps provide a unique opportunity to support Aboriginal children with complex neurodevelopmental or social-emotional needs. Local leadership, combined with a two-way, trauma-informed, strengths-based approach was key to the success of the family camps. This approach could be adopted by similar communities elsewhere.

**Funding:**

This study was supported by a National Health and Medical Research Council of Australia Partnership grant (#APP1171880).

**Research in context**

**Evidence before this study**

Aboriginal Community Controlled Organisations (ACCOs) have led children's health research in the Fitzroy Valley over the past 17 years. In response to a lack of health services and family supports, Marninwarntikura Women's Resource Centre (MWRC) formed the Marurra-U partnership with Royal Far West (RFW). Better understanding partnerships between ACCOs and non-Indigenous health services, alongside any initial impacts of the Marurra-U services is key for improving health and wellbeing of Aboriginal children with neurodevelopmental delays or early life trauma and their families.

**Added value of this study**

This study investigates the design, parent experiences and observations of the Marurra-U family camp. Evidence exists for the benefits of ‘land’ and ‘culture’ camps for Indigenous populations globally. Evidence also exists for the benefits of therapeutic recreation camps for children with chronic illness or disability. This study adds value by investigating the design and potential benefits of therapeutic recreation camp for families with Aboriginal children with neurodevelopmental delays or social-emotional needs. Therapeutic family camps are a locally requested and well received way to support health and wellbeing of children and families in the remote Fitzroy Valley, Western Australia.

**Implications of all the available evidence**

We highlight that the success of the Marurra-U family camps came from combining local knowledge of Aboriginal culture, history, family, and context with clinical knowledge on child development. This led to effective delivery, and enthusiastic uptake, of therapeutic, recreation-based activities and workshops.

## Introduction

Grounded in long-standing systems of cultural governance and collective care, Aboriginal^j^ communities in the remote Fitzroy Valley, Western Australia, are internationally recognised for their leadership in addressing foetal alcohol spectrum disorder (FASD) and supporting families and children with complex neurodevelopmental needs.^1^^,^^2^ Led by senior women whose authority flows from kinship and Country, successive research projects have exemplified Aboriginal self-determination in health research where cultural law guided the scientific process. These research projects have identified and addressed FASD, early life trauma^k^ (ELT),^3^ and developmental delays^4^^,^^5^ among children born in the remote Fitzroy Valley. When local research drew attention to the limited access to health services,^6^ Fitzroy Valley families advocated for increased access to culturally safe health and disability support for children as otherwise children have an increased risk of comorbidities including mental illness later in life.^7^ Our work together highlights the efficacy of community-led partnerships to identify and respond to families’ needs in remote settings like the Fitzroy Valley.^2^ Box 1 outlines the community-led support service described in this paper.

Box 1 **The Marurra-U family camp**

Building on the legacy of community-led innovation, Marninwarntikura Women's Resource Centre (MWRC) formed the Marurra-U partnership with Royal Far West (RFW) embedding a two-way governance model where Aboriginal knowledge and clinical expertise meet on equal ground. The partnership between MWRC, an Aboriginal community-controlled organisation in Fitzroy Crossing, and RFW, a New South Wales (NSW)-based specialist developmental health service implements a wraparound model of care to support school-aged children with complex neurodevelopmental and/or social-emotional needs and their families in the Fitzroy Valley.^2^^,^^8^ This wraparound model is grounded in the Aboriginal understanding of family as a network of kinship and obligation, where wellbeing is collective and healing is relational. As part of the Marurra-U wraparound model of care,^8^ the Marurra-U clinical team (occupational therapists, speech pathologists, social worker) and MWRC family support workers deliver a therapeutic family camp in Broome, Western Australia. The camp is co-led by Aboriginal family support workers and non-Aboriginal clinicians, with an understanding of Aboriginal family and kinship dynamics, particularly in relation to intergenerational trauma, attachment, and bonding.^9^ Using a family-centred approach focused on child, parent, and family outcomes, the camp aims to build children's social, fine and gross motor, and language skills, and emotion regulation through play-based activities^10^^,^^11^ including sport, music, and craft activities. The camp also aims to strengthen children's confidence and personal growth through challenge and risky play^l^.^12^ Through parents and children engaging in these activities together, the camp aims to strengthen inter-family relationships, promote secure attachment, and facilitate shared experiences.^13^ Parent workshops, grounded in the Aboriginal and Torres Strait Islander social and emotional wellbeing model translate the relational and collective nature of Aboriginal wellbeing into everyday parenting strategies, honouring local cultural practice of care while engaging with evidence-based development approaches.^14^ Parent workshops involve parents working on a personal artwork whilst learning about grief and loss, child brain development, and strategies to support children with complex needs. These topics were chosen by the Aboriginal and non-Aboriginal Marurra-U team based on priorities and needs identified through MWRC's research, advocacy and community work, and identified by community members who have previously engaged with Marurra-U services. This family-centred approach resonates with Aboriginal understanding of family as kin-anchored networks of care, where healing occurs through relationship and shared activity. Additionally, this reflects the principle of deep listening and attentiveness to relational obligation, a practice of ethical listening grounded in respect for Country, kin, and story.

Therapeutic recreation camps improve self-esteem, social skills, and social connection in non-Indigenous children with challenging behavioural and emotional needs.^15^ Family camps have been implemented to support children and their families living with chronic illnesses, and improve children's social and emotional functioning^16^; self-efficacy, self-esteem and independence^17^^,^^18^; and health-related quality of life and wellbeing.^17^

Recreation camps also improve parental wellbeing, skills and coping, and social support,^18^ with some positive impacts on family functioning and relationships.^18^, ^19^, ^20^ ‘Land’ and ‘culture’ camps are increasingly recognised as a model of healthcare to promote the wellbeing of Indigenous populations by strengthening cultural identity, increasing social relationships and a sense of belonging, and improving social-emotional wellbeing in Indigenous youth.^21^, ^22^, ^23^, ^24^, ^25^, ^26^

Family-centred support services, which integrate parenting programs with therapeutic interventions for children, support social-emotional development and reduce problem behaviours in children, and improve family functioning, empowerment, and wellbeing in parents.^27^ Family-centred and camp-based programs have been highlighted as appropriate and effective for Aboriginal families in the Fitzroy Valley,^28^ the Northern Territory,^29^ and in New South Wales.^30^ Previous programs have shown improved child behaviours, reduced distress in parents, improved parent-child interactions, and improved parent knowledge of complex needs.^28^, ^29^, ^30^

No previous studies have investigated the implementation and outcomes of therapeutic camps for Aboriginal and Torres Strait Islander families and children with complex neurodevelopmental or social-emotional needs. This research aimed to explore the design, appropriateness, acceptability, and initial parent-reported impact of the Marurra-U family camp through qualitative assessment of parents/carers' and the camp team's experiences and reflections.

## Methods

### Study design

Our Aboriginal-led partnership was underpinned by the principle of relational accountability, recognising community as both partner and authority in research governance.^31^

This study employed an Aboriginal Participatory Action Research (APAR) and strengths-based approach, prioritising Indigenous governance and seeing lived experience as expert knowledge in understanding social and emotional wellbeing.^32^ Strengths-based approaches involved data generation and analysis focusing on what works rather than a deficit discourse, and identifying solutions and actions to achieve family-defined goals. It also involved data generation grounded in contextual and cultural understanding to present nuanced descriptions of family experiences. A strengths-based approach to working with families was also taken by the Marurra-U team, described elsewhere (Pickard et al., unpublished).

Qualitative methods were used to explore the design, appropriateness, acceptability, and initial parent-reported impact of the Marurra-U family camp. This study followed the Australian Institute of Aboriginal and Torres Strait Islander Studies (AIATSIS) Code of Ethics for Aboriginal and Torres Strait Islander Research,^33^ the NHMRC's Ethical Conduct in Research with Aboriginal and Torres Strait Islander Peoples and Communities: Guidelines for Researchers and Stakeholders 2018,^34^ and the Kimberley Aboriginal Health Research Alliance (KAHRA) Principles.^35^ This study is reported according to the Reflexive Thematic Analysis Reporting Guidelines (RTARG) [Supplementary Material 1].^36^

### Setting

Two week-long Marurra-U family camps were held at the Broome Camp School: one in April 2024, another in April 2025. The Broome Camp School is the closest facility to the Fitzroy Valley that provides accommodation, meals and activities at one location. Children and siblings who attended camps were aged 1–18 years. Families who attended the camps were from the remote Fitzroy Valley, located 400 km from Broome. The Fitzroy Valley is home to about 3500 people across 32 communities, of which 80% identify as Aboriginal and belong to five predominant Indigenous language groups: Bunuba, Gooniyandi, Walmajarri, Wangkatjungka and Nyikina.^37^

### Researcher positionality and reflexivity

Researchers who collected and led the analysis of observational and yarning data (AP, TS, SNR, and AM) reflected on their positionality and how socio-cultural, educational, and geographic characteristics may have influenced their interpretation of the data. AP (female) and TS (male) are non-Indigenous Australians, with education and experience in public health research in Australia and Southeast Asia, upbringings in urban locations, and indirect and direct lived experience of disability. AP has experience volunteering at therapeutic recreation camps for children with disabilities in Australia. SNR (female) is a mixed-race Tamil and non-Indigenous Australian with an urban upbringing, education and experience in public health research within Australia, and indirect experience of disability. AM (female) is a dual citizen (Canadian, Australian), with Canadian First Nations family. AM experienced a rural upbringing, has knowledge and experience in public health research globally, lived experience as a family member, of disability, and experience leading and working at therapeutic recreation camps for children and families in Canada, Ireland, Malawi and USA. The research team brings diverse lived experience and worldviews, including Indigenous and non-Indigenous worldviews. Through self-reflection and dialogue with one another, with Aboriginal team members and community members, we have interrogated how our continually shifting identities and worldviews may have shaped our interpretation of data. Additionally, non-Aboriginal team members have each spent between 1 and 15 years working alongside communities in the Fitzroy Valley and have developed strong relationships with community members and Marurra-U team members. Over time, these relationships have deepened their understanding of community contexts, histories, and experiences in the Fitzroy Valley, enhancing their ability to contribute to and present research that is ethically conducted and accountable to these communities.

### Participant recruitment and data generation

Over five days, authors AP (2024, 2025), TS (2024), and SNR (2025) assisted RFW and MWRC to deliver the Marurra-U family camp in Broome. They were involved in all camp activities and conducted ethnographic observations/relational engagement^m^ through notetaking and audio-recording daily debriefs between themselves.

Observations about camp activities, who participated, who facilitated, community leadership, parent session content, staff planning and debrief sessions, and family/camp team interactions were included. Observations were conducted to understand the camp design and dynamic, family engagement, and ways of working in the two-way team. Observations were validated with Aboriginal co-researchers (also members of the Marurra-U staff team) during debriefs at the end of camp, and on subsequent visits to Fitzroy Crossing to ensure cultural accuracy. For example, a non-Aboriginal researcher would note their observations at team discussions, and an Aboriginal co-researcher would validate these observations or provide cultural context and information to put observations in a new light.

On the last day of each camp, JD (an Aboriginal woman from the Fitzroy Valley and an MWRC staff member leading the camp) invited parents who attended the camp to join yarning sessions. In keeping with the aims of the research, and our local ethical approval, JD explained that the purpose of the research was to describe the camp and the experiences of the families, to inform improvements to the program and implementation, as well as understand the services needed by children with complex needs and their families. Camp participants are described in Table 1. All parents/carers, who attended the 2024 camp (n = 7, 100%) agreed to participate in the yarning sessions and at least one parent/carer, from each family (n = 6, 67%) agreed to participate in the 2025 yarning sessions, with everyone providing written consent. 2024 yarning participants were men and women; 2025 participants were all female. Yarning sessions were audio-recorded, ranging from 10 to 40 min [Supplementary Material 2 for yarning session guideline]. Sessions were conducted in English, with further explanation and translation in Kimberley Kriol by JD if required. JD also provided cultural and contextual knowledge throughout the yarning sessions to facilitate shared and accurate understanding between the research team and participants. Audio recordings from the 2024 ethnographic observation debrief session and yarning session were transcribed by a third-party provider (Way With Words Limited, n.d.) and checked for accuracy by AP. The 2025 yarning session recordings were manually transcribed by SNR. Identifiable information was anonymised in transcripts to maintain confidentiality.

**Table 1:** Parent and child demographics.

Participants	2024	Demographics	2025	Demographics
Parents/Carers	n = 7^a^	Female n = 5 Male n = 2	n = 9	Female n = 8 Male n = 1
Children	n = 13	Female n = 4 Male n = 9 Aged 1–18 yrs^b^	n = 12	Female n = 8 Male n = 4 Aged 1–13 yrs^c^

a There was an additional parent on the camp staff team whose children attended camp but was not approached for yarning sessions in 2024.

b Majority of children were aged 5–14 years. One 18 yr old and one 1 yr old sibling attended with their families.

c Majority of children were aged 6–13 years. One 1 yr old sibling attended with their family.

### Data analysis

The 2024 transcripts were inductively analysed using NVivo14 (Lumivero 2024) using reflexive thematic analysis.^38^ AP and TS coded one ethnographic observational debrief and two yarning session transcripts each before meeting to discuss their initial codes and interpretations. AP then coded all the transcripts and constructed sub-themes and themes. AP and TS revised and prepared a final framework of the themes, sub-themes, descriptions, and participant quotes after review by AM. Data were triangulated throughout this process to form a comprehensive qualitative case study. AP then created a visual summary [see Supplementary Material 3] with brief dot points of each theme, and discussed the summary document with JD, VB, and JP (Aboriginal MWRC family support workers, members of the camp team, and co-researchers on the Marurra-U project) in person in Fitzroy Crossing in August 2024. Each person provided feedback on the results and themes, providing further context and approving the document to be shared with participants. Parents and carers who attended camp were then provided this summary in person by AP and invited to provide feedback on the results summary. Two parents/carers had extensive conversations with AP on the summary document, providing further context to the presented results and themes, highlighting important sections, and discussing ways to improve the camp in future. Parents and carers also re-affirmed consent for these results to be presented in this paper.

Themes generated from analysis of the 2024 camp data guided the analysis of the 2025 yarning sessions. This was in line with a reflexive approach, moving between thematic analysis phases and ongoing analysis guiding further investigation.^38^ Reflexive thematic analysis of 2025 data was used to revise and define existing themes, and additional themes were constructed where new concepts or experiences were evident. SNR analysed all 2025 transcripts on NVivo14, while re-listening to recordings of ethnographic observations, and being familiar with themes generated from previous data. AP and SNR then discussed additional and revised themes, and a similar process of community feedback and input on the final themes was followed, as described above, including generating a new visual summary to discuss with Aboriginal co-researchers and camp leaders, and parents/carers in-person in Fitzroy Crossing in September 2025.

### Ethics approval

This study was approved by the Western Australian Aboriginal Health Ethics Committee (HREC1246) and all participants provided informed consent prior to participating in this study. The Kimberley Aboriginal Health Planning Forum also approved this study.

### Role of funding source

Funders had no role in study design, data collection, data analysis, interpretation or writing of the report.

## Results

Four themes were constructed. Relational/observational data and yarns focused on the design and description of the Marurra-U family camp. Parents described what support they sought from camp, their experience, their children's experiences and their children's outcomes in relation to parent goals for their child. Data also described how the Marurra-U two-way team delivered the camp.

### Theme 1. The unique characteristics and design of the Marurra-U family camp

The camps aimed to provide support to children with FASD, ELT, and other complex social-emotional and behavioural needs, and their families. Activities included music sessions, sports, arts and crafts, and other recreation-based activities such as low-ropes courses, swimming and raft building, and parenting workshops. Parent workshops aimed to build understanding of child brain development and behaviour, intergenerational trauma, and grief and loss. Therapeutic aims for children included facilitating connections between children, building vocabulary, speech fluency, oral motor skills, listening and memory skills, and social and expressive language^n^ skills, improving emotion regulation, and social skills, through play-based activities. Parents reported that they were seeking assistance for their children's behaviour, emotion regulation, speech and social skills, with the level of need varying. Family members were also motivated to attend to receive parenting support.

This family-centred approach aimed to strengthen family relationships and child-parent interactions through dedicated time for families to enjoy positive experiences together.

Parents voiced this about camp:

*‘to help him, support him, with his behaviour… having all these strategies, as well, around us, and getting ideas from talking with the… therapist… to work with our children back at home’* [P5, Aboriginal parent/carer 2024].

The camp incorporated local language and knowledge into activities; parents and children taught and led the group in songs in the local Gooniyandi and Walmajarri languages, traditional musical instruments were used in the music sessions, parents taught the team how to make damper, and parent workshops incorporated traditional art. Parent workshops were grounded in the Indigenous social and emotional wellbeing framework,^14^ with parents redrawing the social and emotional wellbeing framework in their own way. Parent workshops were grounded in a trauma-informed, strengths-based^39^ approach. An Aboriginal team member noted this approach from the clinicians *‘make you feel good. They make the families not feel guilty’, ‘there's no judgement’* [Family support worker, MWRC 2024].

Respite was also a strategic focus and outcome of the camp, which enabled parents/carers and children to focus on the camp activities and spend quality time together, away from kinship obligations and day-to-day commitments in the Fitzroy Valley. The camp team reflected that a previous camp held in Fitzroy Crossing was less successful because participants were regularly called away to personal and family obligations. Some parents noted that at home they lacked time to discuss parenting and connect with others in the way they could at camp.

‘*And it’s for…family time when you can sit down, talk*—*talk about your feelings, and…you can make your mind free and like, you know, instead of thinking about the negative things… Got lotta help from everyone and even the good feed I had and—it was so nice to be here, and like when I came, my body just go loose…relaxed*’ [P6, Aboriginal parent/carer 2025].

The camp was part of a broader wraparound model of care developed by the Marurra-U partnership for remote Aboriginal children with complex needs in Fitzroy Valley which included allied health staff engaged in hybrid therapy (in-person and telehealth) with children and providing capacity strengthening for schoolteachers and service providers in the Valley. Most attending families had previous connections with the Marurra-U team through these programs. These parents spoke highly of the Marurra-U team and the improvements they observed among their children, citing this as a motivation to attend camp. Four children who had been receiving allied health therapy via telehealth, and their parents, were able to meet their clinicians in person at the 2025 camp and strengthen their relationships to support ongoing therapy. Post camp, two children were supported with referrals for ongoing assessment, one of which was also supported to gain National Disability Insurance Scheme^40^ access.

### Theme 2. Children actively engaged in camp activities, leading to improved individual, family and social wellbeing

The camp provided children with opportunities to engage in exciting activities and have new experiences individually and alongside their siblings, parents, or other children. One parent described the value of these experiences *‘because it's all different activities held here for these kids, back at home, they don't have activities like that…because hardly, out at community…no playground*’ [P2, Aboriginal parent/carer 2024].

The camp provided children with opportunities for personal growth, including autonomy and problem-solving skills. Participants described their children as being ‘*more confident*’ [P6, Aboriginal parent/carer 2024], and a ‘*bit more independent’* [P2, Aboriginal parent/carer 2025]. One parent noted how their son was ‘*trying to do something by himself*’ [P5, Aboriginal parent/carer 2024]. The children's increased confidence over camp was also displayed through sharing sports and cultural knowledge with others, including the camp team. One parent felt that two-way learning between children and adults (parents and the camp team) empowered children and fostered a sense of pride:

*‘They [the children] were really proud to teach you guys… they felt good within themselves’* [P6, Aboriginal parent/carer 2024]

Parents felt there were behavioural improvements in their children; one noted that their child was more ‘*relaxed and calm*’ [P2, Aboriginal parent/carer 2024] and another recalled that their son ‘*sleeps better [and]…his behaviour is much bette*r’ [P5, Aboriginal parent/carer 2024]. They also noted camp *‘actually helped me figure out to have ways to make him more calmer*’ [P4, Aboriginal parent/carer 2025]. Parents of a child diagnosed with epilepsy perceived a reduction in their son's symptoms throughout the camp, though this was not verified by a health professional and the reason (more consistent medication, decreased stress) was unclear.

‘*He didn't have any seizures at night…with all this activity that was happening all through this week help [him] a lot and even us.*’ [P5, Aboriginal parent/carer]

Camp sports and group activities may have facilitated children's social skills, connection and friendships with others. One parent noted that during camp their son ‘*got more social… because he's really shy and he don't like playing*’ [P3, Aboriginal parent/carer 2024]; with a family support worker adding ‘*he struggles to make friends. He was like, Mum, I made friends with those boys*’ [Family support worker, MWRC 2024]. Children shared positive experiences with their siblings and parents, leading to improved relationships within the family as described by parents. One parent noted that the camp ‘*brought them closer together*’ [P6, Aboriginal parent/carer 2024]. Another parent shared:

‘*Interviewer: you feel like that helped him with his relationship building with other kids? P3: Yes. And especially me, too…he come in the room this morning, he was like, ‘oh, mum, I made you toast’. He done me toast this morning. Interviewer: So your relationship with him, as well, has changed. P3: Yes'* [Aboriginal parent/carer 2024].

Children's engagement with camp activities varied by age, with younger children highly engaged and some adolescents disengaged. The camp team reflected that most camp activities were designed for younger children, and future camps could include activities specifically for older kids, whilst supporting their capacity as role models for younger children. In 2024, the researchers and camp team also observed that children's use of mobile phones during transitions between activities, mealtimes or in the evenings limited opportunities for connection between camp attendees. This was reflected in 2025 parent sessions, which incorporated discussions on managing children's device use; and team members reducing their own phone use during the camp.

### Theme 3. Parents were motivated and empowered to support their children and communities to thrive

Parents sought help for their children's behaviour, speech, emotion regulation, social skills, and how to support their children at home and school. The clinician-Aboriginal staff team at camp shared knowledge about child development, behaviour, and communication, as well as specific strategies to support emotion and behavioural regulation within kinship values and collective care rather than deficit models, which, ‘*with that understanding, it helps us to parent properly, and helps us to understand kids better*’ [P6, Aboriginal parent/carer 2024]. Parents valued knowledge they gained about child brain development and wanted more support to understand and address their children's needs, including for their education and future goals:

‘*Helps us as a parent to understand our children*. *What’s happening with their brain. How can we speak to our children*. *What is the best way for them to learn*. *Because as they get older, we want them to be independent, but we need to find the needs now… for our children to be able to have a stronger education*’ [P5, Aboriginal parent/carer 2024].

In response to families and local Aboriginal staff highlighting the lack of support services, the 2025 parent workshops incorporated discussions around grief and loss, how it can impact children and their behaviour, and how to support themselves and their family.

“*…grief is everywhere in the community… It’s not only when you lose somebody…when you watch other kids upset, you get upset as well, you know? These kids, they know each other… If one is upset, the other will get upset… Everyone in the community is affected by loss and grief”* [P1, Aboriginal parent/carer 2025]

They felt supported through connecting with other parents and by others sharing personal experiences and strategies during workshops:

*“Being able to share with someone else that’s probably going through the same thing or they have a family member that’s going through the same thing as me… this is the most I sit with people, yarning… that was really what I needed.”* [P4, Aboriginal parent/carer 2025]

Some parents suggested that individual family sessions, focused on their child and family needs, would be beneficial. This suggestion aligns with the Marurra-U partnership principle of deep connection in intimate settings where space and time are created for families to speak freely, be heard without judgement, and explore strategies in ways that honour cultural privacy and relational accountability.

Participants and researchers recalled that fathers who attended the camp were actively engaged in parenting workshops and child-bonding activities, particularly sports activities. Participants and MWRC family support workers noted that paternal engagement in these types of activities was not common and saw it as a positive outcome. Participants noted some fathers' engagement was supported by their prior relationship with the camp team through other Marurra-U services. They noted that, in 2025, there were no male staff members. Staff and parents were keen to involve male team members and more fathers and young men in the future to support fathers’ engagement in the camp.

Parents reflected on and applied the knowledge and skills they gained from the parenting workshop to their own experiences as children, parents, and community members. One parent reflected:

*“I had a loss three years ago…and after that, [Child] wanted to be with me all the time… I thought I was doing wrong, you know? By just…keeping him. But, learning from that brain things, and the loss thing that’s been, I’m saying, nah I’m doing the right thing, you know, by listening and today was how even thinking, I’m saying, ‘oh, that’s all about [Child]”* [P2, Aboriginal parent/carer 2025]

Parents valued the knowledge and skills they gained from camp about child development and parenting and wanted child resources to take home and share with their families and communities. Parents knew of other families who would benefit from future camps and felt that having more families attend would improve everyone's experience. They also perceived the knowledge and skills that they gained from the camp would be valuable for school staff to better understand, engage, and support their children:

‘*I wanted to know…about the kids and what we could learn and take back to the school…when we was kids, we had a hard life, and that’s why we going to try change it for the next kids.*’ [P7, Aboriginal parent/carer 2024]

In 2025, the camp provided an opportunity to engage with young adults in community. A young adult sibling attended the parent sessions (rather than engaging in camp as a ‘child’ with their younger siblings) and valued the chance to sit and talk with family. Aboriginal staff members noted the value of this, as young adults are often also viewed as parents in community through the kinship system. One young adult was inspired to pursue further training and employment in child and family support:

*“It’s like nothing back home, you know. And you’re just doing the same thing and circling…I wanna do a, like a something that’s my future, for myself. I wanna like build my confidence up… I would go back to home and tell all my family…for your kids and like, you know, for the future out there… to tell them, you know, pass the message along. There’s just nothing back home"* [P6, Aboriginal parent/carer 2025]

### Theme 4. Two-way teams are effective in delivering child and family support

The Marurra-U two-way teams integrated Aboriginal and Western knowledge^31^ to engage with Aboriginal children and parents/carers in a culturally safe, relatable, and practical way. This approach also fostered a sense of pride in the community:

‘*I'm really proud because [Name], she knows the community very well, she can speak in our own language where we can understand her… and who can explain much better. And you also have [Name] there because…she’s professional at what she do. Them two working together makes a big difference because it’s coming out from an Aboriginal woman too’* [P5, Aboriginal parent/carer 2024]

Participants noted that the professional knowledge and non-judgemental approach, combined with a deep understanding of the local context, was key to the success of parent workshops. Aboriginal team members were able to connect clinicians' knowledge on child brain development, trauma, behaviour and communication, and grief and loss, to communities' and families’ experiences, cultures and histories. This facilitated two-way learning within the camp team, and between the team and parents. Aboriginal staff could also acknowledge and explain the impact that colonisation and intergenerational trauma have on parenting and family functioning in the Fitzroy Valley.

An Aboriginal team member noted that camp presented a new way of parenting that was not commonly seen in local cultures. They highlighted that what may be perceived as ‘low engagement’ from parents in some activities is related to ongoing effects of intergenerational trauma that have impacted Aboriginal family structures and interactions, cultural differences in parenting styles and relationships, and parents reflecting and processing new information. This observation was supported by one parent recalling *‘it's another new whole step for me, as a mum, as a single parent, as well, and knowing all these new stuff, it's really hard and new to me’* [P1, Aboriginal parent/carer 2024]. However, parents did actively engage in parent sessions, listening and taking notes, and some joined activities such as playing sport, singing, jumping in the pool or doing craft. Despite cultural differences, parent engagement alongside their children was seen as a positive outcome by both Aboriginal and non-Aboriginal team members and family members. Two-way teams built this engagement in culturally safe ways, particularly regarding the team's role-modelling approach to facilitate parent-child interactions. The 2025 camp staff included some Aboriginal parents who had attended the 2024 camp. The camp team considered this a key influence in increased parent engagement in 2025, facilitating trust between the non-Aboriginal staff members and parents:

“*I don’t leave any of my kids with anybody but my parents…even when I go to the hospital…you guys were so nice…that made me open up to allowing you guys, trusting you guys to watch my other kid, all my kids… that’s when I said, my kids will be—my kids are safe*” [P4, Aboriginal Parent/carer 2025]

Parents noted the difference between this relationship with the two-way team compared to other health services they had previously accessed:

“*A week is…a good while…taking more time to get to know them and get things done and visually see what’s happening with them. Whereas, other services just there for a couple of hours and… they don’t actually know what you mean sometimes…being at a camp, you’ve got that time to see and listen*” [P1, Aboriginal parent/carer 2025]

## Discussion

In this qualitative study we explored the design, appropriateness, acceptability and initial parent-reported impact of the Marurra-U family camp for children with complex neurodevelopmental or social-emotional needs and their families. The results demonstrate that a community-led, two-way (Aboriginal and non-Aboriginal) team was successful in delivering a therapeutic, recreation-based camp tailored to the social and historical context of families attending. They show a positive initial impact on family relationships, parent knowledge and ability to respond to child needs, and on children's social-emotional wellbeing and social skills, as described by parents.

Parents and carers attending the Marurra-U family camp described improved relationships with, and understanding of, their children. Parents also reported an increased understanding of strategies to support their children's social-emotional wellbeing and emotion regulation at home following community and professional co-led parent workshops on child development, managing challenging behaviour, parenting, and grief. This suggests that positive outcomes, meeting parent voiced needs, may be possible to achieve through the family-centred, therapeutic camps. Our findings align with literature on family-centred interventions to support child development through strengthening child-parent relationships, shared parent-child activities, and improving parent capacity to respond to children's needs and behaviour.^41^^,^^42^ Our findings also align with literature on parent training programs with Aboriginal and Torres Strait Islander families, which have demonstrated positive impact on children's behaviours and parenting strategies through focusing on parent training, supporting child social skills, and strengthening parent-child interactions.^28^^,^^29^^,^^43^ The Marurra-U camp implements recent recommendations to integrate kinship systems of support with health services support and upskilling community members to implement strategies learnt from health professionals.^7^ In Aboriginal contexts, shared parent-child experiences are also acts of cultural renewal, moments where kinship ties, reciprocity, and belonging are reinforced through being and doing together. Our findings highlight that when Aboriginal families are supported to lead in their children's development, the work moves beyond service delivery to become an act of governance.

Our findings showed that children benefited from the social nature of the camp and built new friendships. This aligns with previous studies on child and family-centred recreational camps and suggests this format can support child social skills.^15^, ^16^, ^17^, ^18^^,^^44^^,^^45^ These friendships also reflected the cultural significance of social connection in Aboriginal childhoods. Through play, language, and shared activity, children were re-establishing the kinship networks that sustain wellbeing and identity. Social development, in this sense, was not only interpersonal but also cultural, grounded in belonging to each other and to Country. Parents described children as more independent, confident, and calmer. Similarly, previous literature on recreational camps have shown improvement in children's self-efficacy, self-esteem, and autonomy.^17^^,^^45^ A family support worker noted that attending camp with siblings and other kids, where all children were completing activities together, may have contributed to children with complex needs feeling confident in themselves, and not singled out as *‘the one who needs help’* or *‘different’*. Parent-reported outcomes of children being calmer may be due to improved social-emotional functioning as reported in previous studies.^16^ Additionally, increased calmness may be due to respite from stressors such as home overcrowding or lack of sleep, increased feelings of connection or safety with parents facilitated throughout camp, or a reduced need for hypervigilance or need to gain parents' attention through less acceptable means.^46^ Moreover, increased time engaging in physical activities outdoors has been shown to increase calmness in children.^47^

It was noted that children's engagement in the camp differed by age, with some of the older children less engaged. Previous studies of therapeutic recreation programs in other settings indicate that increasing opportunities for independence for older children attending the Marurra-U camp and supporting connection to Elders could lead to more positive engagement of this cohort.^24^^,^^48^ MWRC has previously led research into adolescent and young adult social and emotional wellbeing support services, and there is opportunity to build this learning into the Marurra-U family camp.^49^ Additionally, measures to limit phone use during the day could be discussed for future camps. Paternal engagement in the camp was a strength, however, there is opportunity to build this further, as desired by parents and the camp team. Including fathers in parent training programs can lead to greater improvements in child behaviour outcomes^27^^,^^50^ and has been hypothesised as a mechanism for further improving social and emotional wellbeing outcomes for Aboriginal and Torres Strait Islander boys.^29^

The study revealed the two-way camp team was successful in building positive relationships and trust with parents and carers. Two-way teams have previously been identified as key mechanisms of success in parenting support programs with Aboriginal families.^30^^,^^43^ Although previous land or culture camps for Indigenous populations differed in content and structure across communities and settings, leadership from Elders and Knowledge-keepers, and intergenerational connections were found to be strengths of such programs.^21^, ^22^, ^23^, ^24^, ^25^^,^^51^ When therapeutic approaches are led and owned by community, guided by Aboriginal ways of knowing, being and doing, they support both healing and self-determination. Previous literature into family-centred support highlights improved service outcomes when positive relationships between service providers and parents are present, particularly in Aboriginal and Torres Strait Islander settings.^41^^,^^52^, ^53^, ^54^, ^55^ Relationship-based models align with Indigenous worldviews and are an important aspect of ‘decolonising’ healthcare services.^7^^,^^56^, ^57^, ^58^ Strengths-based approaches aligned with Indigenous views of disability further contributed to successful relationship building between staff and families.^7^^,^^59^ Providing support alongside everyday activities and allowing parents, clinicians and local Aboriginal family support workers to see interactions in a naturalistic setting was an important aspect of the Boomerangs family camp,^30^ found to align with Aboriginal knowledge sharing methodologies, and may have contributed to the success of parent support during the Marurra-U family camp. Two-way teams were vital to understanding parent engagement in camp by taking into consideration cultural differences and the impact of intergenerational trauma on Aboriginal families and parenting structures. Two-way teams could be further utilised to strengthen parent engagement in the camp or other health services through culturally safe support. This includes practical support for clinicians such as language translation, or understanding whether speech delays are present, or a reflection of speaking in a second language.

The study highlighted the importance of using a trauma-informed approach based on understanding of local history and culture to achieve outcomes in this context. Previous studies have explored the effects of colonisation and trauma on parenting in Aboriginal families including impact on attachment relationships between children and carers, loss of parenting roles and skills, family functioning, and disconnection from culture and society.^9^^,^^60^^,^^61^ Improving health and social outcomes for Aboriginal Australian families requires recognising the intergenerational resilience, cultural continuity, and collective strength that have carried communities through the ongoing impacts of colonisation, displacement, forced child removals and racism, while supporting culturally grounded pathways to healing and wellbeing.^60^^,^^62^ Previous research with Aboriginal parents sharing successful parenting strategies they use to ‘break the cycle of trauma’ includes focussing on history and education, healthy culture and community, healthy parenting, and a healthy mind (parents looking after their own wellbeing).^61^ The importance of supporting Indigenous parents affected by trauma was also noted by participants in the Jandu Yani U program in the Fitzroy Valley,^43^ where present-day disadvantage and intergenerational trauma can significantly impact parenting, personal coping and self-regulation skills.^28^ Beyond recognition of the ongoing impacts of intergenerational trauma, a trauma-informed approach to working with families involved meeting parents and kids where they were at, and walking alongside them to achieve their family goals. A focus on trauma-informed practice, parent support, and cultural understanding of Aboriginal parenting and how colonisation has affected this was a strength of the Marurra-U camp and this approach within the Marurra-U model of care has been described elsewhere (Pickard et al., submitted).

The other camps specifically for Indigenous populations evaluated in the literature are often conducted on-country and are focused on connection to culture and Country as the main outcomes and method for increasing wellbeing.^21^, ^22^, ^23^, ^24^, ^25^^,^^63^ These are often implemented in colonial settings where Indigenous participants have lost connection to Country and camps are able to facilitate spending time on-country and fostering this connection. Some families attending the Marurra-U family camp live on or near their traditional lands, with connection to Country not forming a key goal of the Marurra-U family camp. Our findings suggest holding the Marurra-U family camp away from the attendees’ homes in the Fitzroy Valley, apart from their family and community obligations or distractions—enabled families to focus on camp activities and building connections. We found that the Marurra-U camp provided families with respite, which is often cited as a positive parent impact of recreation camps for children with chronic illness.^17^, ^18^, ^19^ While the Marurra-U camp included songs in local languages, and tied discussion to local histories and cultures, camp aims focused more on parent knowledge, child behaviour and family connection outcomes. Existing evidence suggests including local stories, integrating traditional healing practices or ceremonies, or including more cultural recreational activities led by Aboriginal team or family members may improve outcomes even further.

Most families who attended the Marurra-U family camp had engaged with the broader wraparound services offered by the Marurra-U partnership. A systematic review of therapeutic recreation camps showed follow-up services and supports maintained and increased the impact of such camps, and that camps could act as an outreach method for families to join additional support services.^18^ This is encouraging for the Marurra-U family camp as it is part of a broader model of care, where follow up services for families can increase the impact of the Marurra-U camp.

### Limitations

This study had a number of limitations. Yarning sessions only included parents and carers, so children's voices were missing. The number of participants was small and the age of children and their disability varied. No quantitative measures were used, however, this was in line with Indigenous research methodology of APAR. Longitudinal data and further research is needed to explore potential specific child outcomes and secondary effects on school performance or educational outcomes, especially in the context of a broader model of care. Whilst this qualitative study suggested promising impacts in social and personal or family level outcomes as felt by parents, specific therapeutic outcomes such as language or motor skill changes were not captured through formal assessment. These remain goals of the wraparound model of care and there is opportunity here to develop culturally appropriate evaluation tools for such models. Few tools exist to holistically capture impacts from wraparound models, tailored to Indigenous ontologies and epistemologies. Future research could involve measures of success co-designed with families and community members that reflect community priorities in healthcare, community identified indicators of social-emotional wellbeing, and indicators of culturally safe care.

### Conclusion

Family-centred, therapeutic, recreation-based camps provide a unique opportunity to support Aboriginal children with complex neurodevelopmental or social-emotional needs. They do so not only by offering structured therapeutic activities, but by creating culturally governed spaces of belonging, where families can reconnect through Country, story, and shared experience. Within these environments, healing is relational, emerging from trust, kinship, and the authority of community leadership and expertise as much as from clinical expertise. Key success factors include two-way teams, with parent support led by local Aboriginal women, and camp activities tailored to suit the local context, histories, and cultures. These features embody Aboriginal governance in practice: leadership through women's cultural authority, decisions grounded in kinship responsibility, and learning that occurs through doing, yarning, and caring together. Two-way teams make visible the balance of knowledges—clinical and cultural—ensuring programs are guided by community law and relational ethics rather than external frameworks. Using a trauma-informed approach, and recognising the impact of colonisation, intergenerational trauma, and ongoing challenges such as poverty, grief, and loss were vital to working with families in a strengths-based and culturally safe way. Respite for parents was a key factor in parent engagement and wellbeing in the program. The Marurra-U family camp showed promise as a support service to improve child, parent, and family outcomes, including emotion regulation, social skills, confidence, and behaviour in children, and empowered parents with increased knowledge and skills to support child development. The Marurra-U family camp is a positive component of a broader wraparound model of care providing telecare to children, and capacity building to service providers in the Fitzroy Valley.^8^

The Marurra-U partnership is a weaving–strands of culture, community and clinical knowledge interlaced through respect. Its strength lies not in one thread but in how they hold together under tension. What might our health and research systems look like if every program were woven in this way, through Aboriginal leadership, trust, and relationship?

## Contributors

AP (non-Indigenous): Conceptualisation, investigation, data curation, formal analysis, methodology, project administration, validation, visualisation, writing–original draft, writing–review and editing. TS (non-Indigenous): Conceptualisation, investigation, data curation, formal analysis, methodology, project administration, validation, writing–original draft, writing–review and editing. JD (Aboriginal, Gooniyandi and Kija): Conceptualisation, funding acquisition, investigation, methodology, resources, validation, writing–review and editing, and contributed Aboriginal knowledge and lived experience. SNR (non-Indigenous): Investigation, methodology, formal analysis, data curation, project administration, writing–review and editing. EC (Aboriginal, Gooniyandi and Kija): Conceptualisation, funding acquisition, supervision, and contributed Aboriginal knowledge and lived experience. ST (non-Indigenous): Conceptualisation, funding acquisition, methodology, resources, writing–review and editing. VB (Aboriginal, Gooniyandi and Walmajarri): Resources, validation, and contributed Aboriginal knowledge and lived experience. ZJ (Aboriginal, Jaru): Resources, validation, and contributed Aboriginal knowledge and lived experience. JP (Aboriginal, Gooniyandi and Walmajarri): Resources, validation, and contributed Aboriginal knowledge and lived experience. CH (non-Indigenous): Conceptualisation, methodology, resources, writing–review and editing. RE (non-Indigenous): Conceptualisation, methodology, writing–review and editing. KC (non-Indigenous): Conceptualisation, funding acquisition, resources, writing–review and editing. TE (Aboriginal, Noongar and Yamatji): Funding acquisition, writing–review and editing, and contributed Aboriginal knowledge and lived experience. EE (non-Indigenous): Conceptualisation, funding acquisition, supervision, writing–review and editing. AM (Inuit Family): Conceptualisation, funding acquisition, methodology, resources, project administration, validation, supervision, writing–review and editing, and provided Indigenous knowledge and lived experience. AP, TS, and SNR accessed and verified raw data. Data were further verified by AM, JD, VB, JP, ZJ, and RE. All authors were responsible for the decision to submit the manuscript.

## Data sharing statement

The data is owned by the communities of the Fitzroy Valley, facilitated by MWRC and are not publicly available due to confidentiality and security. Access to datasets is provided by MWRC and The University of Sydney (contact: Alexandra Martiniuk, alexandra.martiniuk@sydney.edu.au).

## Consent to publish

Not Applicable.

## Declaration of interests

ST, EC, and JD are employees of MWRC and CH, KC and RE are employees of RFW. AP, TS, and SNR were supported through NHMRC to travel for data collection to Fitzroy Crossing (#APP1171880).

## Footnotes

l Risky play describes play activities that require children to understand situations, assess their own capabilities, test limits and manage risk, important for child development.^12^

## Footnotes

j Note that hereafter, the term “Indigenous” will be used in an international context, “Aboriginal and Torres Strait Islander” refers to the First Peoples of Australia and the people of the Fitzroy Valley in the Kimberley region in Western Australia will be described as “Aboriginal” according to their preference.

k Adverse childhood experiences (ACEs) and ELT are closely linked. This team conceptualises that ACEs are events that can increase chances of experiencing ELT and use the term ELT as such in this paper.

m Ethnographic observation is widely understood as a Western research method that has historically enabled research to be conducted on Indigenous peoples rather than by Indigenous researchers. Guided by an Indigenous research paradigm and APAR, the approach used in this study instead acknowledges knowledge as emerging through collaboration, dialogue, and the development of relationships.^31^ The research team actively participated in partnership activities, observing, engaging, and learning alongside participants and co-researchers. Knowledge was therefore produced through shared participation and collective experience, rather than through detached, ‘objective’ observation. We describe this approach as ethnographic observation/relational engagement to emphasise how it differs from conventional ethnographic observation methods.

n Note that while clinicians speak and practice in Standard Australian English, they work in tandem with MWRC family support workers to discuss if language difficulties observed in English are present in Kriol or traditional languages, and to tailor speech support accordingly.
