# Supplementary material for: Family-centred, therapeutic camps for Aboriginal children with complex neurodevelopmental needs and early life trauma in remote Australia: a qualitative study of the Marurra-U family camp
Source: Lancet Reg Health West Pac. 2026 Sep 10;74:101974. doi: 10.1016/j.lanwpc.2026.101974 (PMC13584108; doi:10.1016/j.lanwpc.2026.101974)
Supplement: Supplementary file 1 [file mmc1.docx]

Supplementary Material 1 – Reflexive Thematic Analysis Guideline

| **Advice for aspects of the**  **research report/approach to**  **reporting** | **Guiding notes and further**  **explanation** | **Practices, concepts and**  **terminology to avoid** | **Our Notes** |
| --- | --- | --- | --- |
| The Introduction  NB: We prefer Introduction over Literature Review as a section heading, to capture the broader  purpose of this section. | | | We begin with ‘Introduction’ p.4 |
| Background and rationale | | |  |
| Provide a robust context and  rationale for the proposed  research in the Introduction. | Can discuss existing research,  theory, and the wider context;  the researcher is understood  as entering a conversation  with existing scholarship. | Critiquing the methodological  limitations of existing research  from a (post)positivist/  quantitative standpoint;  orienting a literature review to  finding a “gap” that the  research fills. | p. 4-6 |
| Clearly articulate a research  question – one that is  methodologically coherent. | Can discuss refining an initially  broader research question to  a more specific one for the  paper. | Formulating research questions  as hypotheses or expectations  about what might be “found”. | p. 6 clear research aims are stated. |
| “Owning your perspectives” | | |  |
| Include information on guiding  theoretical assumptions and  other (e.g., explanatory) theory informing the use of  TA. | Guiding (e.g., paradigmatic,  ontological and  epistemological) and other theory should be coherent  with RTA. | (Post)positivism and (simple)  realism. | Aboriginal participatory action research outlined in study design p 7. |
| Report in a way that is  consistent with stated  theoretical assumptions  throughout. | Theoretical coherence is  evidenced through the use of  language and concepts (e.g.,  around theme development,  research subjectivity, data  interpretation), the treatment  of data, and use of quality  practices consistent with RTA. | Inadvertently “mashing-up” of  RTA and (post)positivism/  realism (e.g., assuming data  interpretation can be accurate  and reliable) – without a clear  rationale. | Done. APAR alignment described p. 9 and evidenced throughout. |
| Evidence methodological  coherence/integrity in both  the research and the report. | Theoretical assumptions,  research questions,  methods/practices of data  generation, RTA, and specific  orientation to RTA, purpose of  research etc. all “fit together”,  conceptually. | Ontological and  epistemological confusion (e.g.,  claiming constructionism but  focusing on lived experience  and treating language as a  transparent window onto this). | p. 7 - 11 |
| Show evidence of reflexive  practice. | Can discuss researcher  professional or personal  positioning and experience in  relation to the topic, and/or  participant group, and/or their  role in shaping the research;  use of reflexive journaling. | Evoking researcher bias  (positivist), or even researcher  influence, in a way that evokes  it as possible rather than  inevitable. | Reflexive statements included p. 7 -8. |
| Write in a methodologically  coherent style. | A first-person writing style  suits RTA, as it “writes in” the  researcher and contributes to  situated and reflexive  reporting. | A third person writing style –  writing the researcher out of  the research. | Done. |
| The Methodology  NB: We prefer the theoretically-embedded term Methodology as a section header, over the  proceduralist term Method. | | | Note we have used ‘methods’ to align with journal formatting requirements. |
| Participants/data items |  |  |  |
| Describe selection of  participants/data items. | Should include criteria for  selection and/or recruitment  strategies and settings. | Terms “sample/sampling”,  which connote “sampling” from  a population (for the purpose  of statistical generalisation). | p. 8-9 |
| Describe number of  participants/data items;  provide a rationale or  explanation around dataset or  participant group  size/composition. | Non-positivist qualitative  concepts, such as  “information power” or  sufficiency offer conceptually  appropriate justifications for  “dataset” or “participant  group” size and composition.8 | Justification based on  saturation (simple realist), or  statistical models (positivist);  reporting rates of non-  participation (an indicator of  the representativeness of the  “sample” in quantitative  research). | p. 9 |
| Discuss characteristics of  participants/data items. | Balance the need to “situate  the participant group” with  participant anonymity (e.g.,  aggregate or report minimal  demographics where  appropriate).  6 | Tables with each participant’s  demographic information listed  line-by-line. | p. 8-9, table 1. |
| Detail ethical approval and  ethical code/principles  followed, participant informed  consent, etc. | Ethical discussion usually  includes institutional ethical  approval (if needed), but may  include wider principles;  providing research materials  (participant information,  consent form, etc.) in  supplementary materials may  be useful to support reflexive  openness. | Compromising participant  anonymity by the details  provided. | Informed consent p. 9, p. 11, ethics p. 11 |
| Dataset generation  NB: We prefer the term generation over collection to capture the active role of the researcher and  that data don’t pre-exist research as data, but become data through research practices. | | | We have used generation throughout. |
| Provide some rationale for  method(s) for data  generation/data item sources  chosen. | Discuss why the method(s) of data generation/data source  was a good fit with the  research question, participant  group, guiding theory, etc. If  multiple data sources are  used, any rationale for  combination should be  conceptually appropriate (e.g.,  crystallisation). | Triangulation as a rationale for different data sources (realist). | p. 9 |
| Describe development and/or  characteristics of data  generation tool(s). | Include tool(s) in  supplementary materials  when possible; discuss piloting  if used, and any changes  following piloting, or during  data generation. | Using an existing tool with the  aim of replicating existing  “findings”, or developing and  describing a tool in a way that  is intended to facilitate future  replication (positivist). | p. 9-10, supplementary materials 2 and 3. |
| Include details such as  modality and/or setting of  data generation, time frame,  and other pertinent  procedural information. | Relevant information includes:  the mode of a data generation  tool (e.g., video call focus  groups; chat-based  interviews); the context of  data generation (location;  timeframe) – where this  doesn’t compromise  participant anonymity; and  mode of recording interactive  data generation. | Standardisation as a gold  standard (realist); justifying an  aimed for standardisation in  data generation tools as a  means to facilitate the  “reliability” or “accuracy” of  the research; treating a lack of  standardisation in data  generation method, modality  or setting as a problem, a  potential source of “bias”. | p. 9 |
| Describe who conducted any  interactive data generation  (which author or research  role), and how. | Can include what, if anything,  the researcher disclosed about  their personal or professional  positioning or motivation;  what skills and experience  they brought; note  researcher’s relationship with  participants prior to, during  and after the research. | Seeking standardisation (e.g.,  through the training of  researchers) in interactive data  collection; treating non-  standardisation as a threat to  “reliability” or “accuracy”. | p. 8-9 |
| Describe the size/scope of  dataset and dataset items. | Such as the range and average  length for interviews/focus groups; range and average word length for textual data  items. | Equating data quantity with  data quality. | p. 9 |
| Describe, and if relevant  explain, any preparation of  data for analysis. | Such as method of  transcription of audio/video  data (a transcription key can  go in supplementary  materials); changes and  “corrections” – such as why  typographical errors in written  data were corrected; system  for removing any identifying  information; use of  pseudonyms and/or data  codes. | Describing transcription as  “verbatim” or “orthographic”  with no further details; using  edited or “cleaned up” data  without acknowledgement of  this; participant validation of  the “accuracy” of transcripts  (realist). | p. 9 |
| Data analysis | | |  |
| Provide some rationale for use  of RTA, and, where relevant,  for combining RTA with other  approaches and procedures. | Any combining of RTA with  other method/ologies or  procedures should be  warranted, rather than based  on a misunderstanding of RTA,  and conceptually coherent  (unless clearly justified). | Citing generic characteristics of  RTA (e.g., accessible, flexible)  without explaining how they  were relevant to the study;  using a codebook without  acknowledging this is not part  of RTA and justifying its use. | p. 10 |
| Describe specific orientation  to RTA. | Locate RTA on dimensions of  inductive<>deductive and  semantic<>latent. | A generic discussion of TA (or  even RTA), not specifically  situated in relation to the study  or approach. | p. 10 |
| Discuss how the researcher(s)  engaged with the analytic  process. | Provide a specific and situated  account of the analysis  process; use supplementary  materials to provide a fuller  account of the analytic  process. | Offering a generic description  of the six phases of RTA in lieu  of an account of analytic  process. | p. 10 |
| Where more than one person  is involved, describe who  analysed the data (author or  research role). | Role(s) or involvement  throughout the process should  be discussed; where coding  was collaborative, what this  involved and how differences  in coding and theme  development were tackled,  should be included. | Use of inter-coder agreement  measures, consensus coding  approach (positivist). | p. 10, p. 25-26 |
| Use language to describe the  process and products of RTA  that is coherent with the  values and assumptions of  RTA. | Language should convey the  active role of the researcher(s)  in “generating”  ,  “crafting”  ,  “constructing”  ,  “creating”,  “producing” or “developing”  themes; language around  themes should evokes them as  products of a researcher-data  process. | Passive language of discovery,  such as “emerging”, “found”,  “identified”, “discovered” –  these evoke themes as  “diamonds scattered in the  sand” (p. 740)10; unexplained  use of language and concepts  from other approaches, such as  emergent or superordinate  themes (IPA), or line-by-line and/or open coding and  constant comparison  (grounded theory). | p. 10, p. 11-18 |
| The Analysis  NB: We prefer the heading Analysis over Findings/Results. Findings implies the researcher  “found”, “discovered” or “identified” pre-existing themes. Results is strongly associated with the outputs of statistical analysis. | | | Note we use ‘results’ to adhere to journal formatting requirements |
| Reporting the data analysis | | |  |
| Provide an overview of themes or thematic structure. | Overviews can include a list, map or table of themes to  preview the analysis. | An unclear thematic structure, including unexplained headings  in the Analysis. | p. 11 |
| Ensure theme  conceptualisation is  appropriate to RTA, and any  divergences are justified and  explained. | In RTA, themes report shared  meaning, united around a  central organising concept  that differs for each theme. | Topic summaries; data  generation questions reported  as “themes”. | p. 11-18 |
| Name themes appropriately. | Use theme names that  capture the “essence” or  “story” of each themes; brief  data quotations can be used. | (One-word) theme names that  only identify a topic, and offer  no story (evoking topic  summaries). | p. 11-18 |
| Report themes in sufficient  depth and detail. | As RTA is an interpretative  method, themes should be  multifaceted, and contain  both data and analytic  narrative; if useful, additional  data extracts may be included  in supplementary materials.  7 | Thin, one dimensional themes,  effectively conflating codes and  themes; large number of  themes relative to the length of  the manuscript. | p. 11- 18 |
| Use subtheme judiciously. | Themes are the main analytic  purpose, and should be  multifaceted; only use  subthemes where doing so  highlights an important facet  or aspect of the central  concept of a theme. | Fragmenting the analysis  through overuse of subthemes,  and an overly  elaborated/“bitty” thematic  structure. | No subthemes used. |
| Ensure the analytic narrative  explains the meaning and  significance of the data. | For RTA, each theme needs an  analytic narrative that outlines  its meaning and importance in  relation to the topic, research  question and dataset; the  reader needs to be told about  why/how data excerpts  matter and “evidence” the  theme; the Analysis section  also needs to convey the  overall story of the analysis. | Frequency counts as a  justification for themes  presented; simple paraphrasing  of data as “analytic narrative”;  treating data meaning as self-  evident (data are assumed to  speak for themselves);  “arguing” with the data  (treating the data as something  to [dis]agree with, rather than  to interpret and make sense  of). | p. 11- 18 |
| Provide an appropriate  balance of analytic narrative  and data extracts – both data  extracts and analytic narrative  matter. | The rich descriptive and/or  interpretative story of the  analysis needs to be woven  around sufficient analytic extracts from across the  dataset. | Presenting either a long string  of data extracts with barely any  analytic narrative, or only the  researcher’s narrative summary of the theme, without any data  extracts to support it. | p. 11 - 18 |
| Demonstrate coherence  between analytic narrative  and illustrative/evidentiary  data extracts. | Data extracts should  convincingly and compellingly  evidence the analytic claims. | Mismatches between data  extracts and analytic claims;  not countering obvious  alternative readings of the data | p. 11 - 18 |
| Integrate existing research  and theory into the analytic  narrative. | In RTA, an interpretative  analytic narrative is enriched  by incorporating relevant  existing research and theory  into the reporting of themes,  reflecting notions of  contextualised meaning, and  contributing to an ongoing  “conversation” about a topic. | The positivist tradition of  separating a description of  analytic “Results” and their  interpretation with reference  to scholarship and theory in a  “Discussion” section. | Following conventions of the journal and health discipline the results and discussion are presented separately though analysis in the discussion blends existing research and results. |
| The Final Section – A General Discussion or “Conclusions”  NB: We don’t have a preference for what a final section of an RTA report is called, and it depends  on the context and the focus and purpose of the study – the heading Conclusion may evoke a  certainty that isn’t appropriate; Implications may be useful; Final Considerations or Reflections  may work, as might General Discussion. | | |  |
| Quality, evaluation and conclusions | | |  |
| Draw analytic conclusions  across themes. | Orient to the “so what” of the  overall analysis – the “point”  of the story told; this might  include discussion of  implications for practice and  “actionable” outcomes. | Repetitive theme-by-theme  integration of the analysis with  existing literature; no overall  conclusions drawn; no overall  analytic story. | p. 19 - 22 |
| Discuss implications or  directions for future research. | Any suggestions for future  research should stem from the  analysis and be evidence-  based (e.g., provide grounds  for other groups potentially  having different experiences  or views) rather than generic. | Generic recommendations for  other research, such as with a  different “population”. | p. 23 |
| Use and report quality  practices coherent with RTA. | Ensure evaluation of research  quality deploys conceptually  coherent notions, such as:  member reflections;  crystallisation;9 others serving  as a critical friend/sounding  board to enhance insight;12  reflexive journaling. | Incoherent quality measures  such as: member  checking/participant validation;  triangulation (realist); the use  of theme agreement/consensus  among researchers or  corroboration of themes by  another researcher (positivist). | Supplementary material 1. p. 8. |
| Evaluate the research from a  Big Q standpoint. | Such evaluation might  including considering how the  specifics of the study may  have shaped the research  produced (for example, the  characteristics and context of  the participant group/dataset;  the methods and modalities  for generating the data);  situatedness should not be  treated as a limitation | Evaluations and descriptions of  limitations that orient to  quantitative or positivist  norms, such as reference to  lack of generalisability –  positioned as a limitation, and  equated only with statistical  generalisability13  – or a “small” (by implication non-ideal) and “unrepresentative” sample. | p. 20, 23, |
| Include reflections on  research process and  practices, including researcher  reflexivity. | Some consideration of the  researcher(s)’s role in shaping  the research and the  knowledge generated is an  important quality marker. | Reference to researcher  bias/influence (positivist). | p. 7 - 8 |
