# Supplementary material for: Family-centred, therapeutic camps for Aboriginal children with complex neurodevelopmental needs and early life trauma in remote Australia: a qualitative study of the Marurra-U family camp
Source: Lancet Reg Health West Pac. 2026 Sep 10;74:101974. doi: 10.1016/j.lanwpc.2026.101974 (PMC13584108; doi:10.1016/j.lanwpc.2026.101974)
Supplement: Supplementary file 2 [file mmc2.docx]

**Marurra-U yarning session guideline**

Participants:

Parents engaged in MWRC and Royal Far West’s camp in Broome, Western Australia in 2024.

Introduction:

The NHMRC Marurra-U Project is based on a longstanding partnership between Marninwarntikura Women’s Resource Centre (MWRC), Royal Far West (RFW) and the University of Sydney (USYD).

This partnership aims to ensure that children and families in remote Aboriginal communities in the Kimberley have access to a connected system of supports that are strengths-based, grounded in local wisdom, and focused on enabling children to develop the capabilities they need to participate meaningfully in all aspects of their life.

I am working for the NHMRC Marurra-U Partnership to improve these supports. I would like to ask you some questions about your involvement in the camp in Broome and/or other support services provided by RFW and MWRC in Fitzroy Valley.

Please tell me if you would like to take a break or stop this session.

*START INTERVIEW RECORDIING*

Topic 1: introductions

- A community navigator will introduce the session and research team member (University of Sydney)

- Research team member (University of Sydney) shares his background

- Invite others to share their own stories

Topic 2: camp in Broome and/or other support services provided by RFW and MWRC

- Research team member shares how he came to be involved in the camp in Broome and/or other support services and his experience

- Invite others to share their stories of how they came to be involved in the Broome camp and/or other support services provided by RFW and MWRC, including what they hope to gain from this camp.

Topic 3: Experience of the camp in Broome

- Research team member shares his most memorable experience of joining the camp in Broome

- Invite others to share their most memorable experience of the camp in Broome

- Invite participants to share their most memorable thing they learned from the camp in Broome and how it impacted them, including how they have used and/or plan to use the knowledge, skills or relationships they gained from the camp

- Ask participants to share if they noticed any changes in their children’s social-emotional well-being or behaviour from using this experience, knowledge, skills or relationships or think will happen if they use this new experience, knowledge, skills or relationships.

- Invite participants to share what they noticed about their children’s experience of attending the camp, including things they learned, memorable moments, and/or changes in their knowledge, attitudes (thoughts/comments) or actions (e.g. behaviour).

Topic 4: Improving the camp and other supports/services

- Invite participants to share their thoughts on how the camp or other support services could be improved

- Invite participants to share their thoughts on what other supports/services they might find helpful to supporting their children in school and elsewhere

Conclusion

- Invite participants to share what they think this research and the NHMRC Marurra-U Project could do to support them and their communities better.

Final questions/comments

- Invite participants to ask or make any final comments.

*STOP INTERVIEW RECORDIING*
