# Supplementary material for: Family-centred, therapeutic camps for Aboriginal children with complex neurodevelopmental needs and early life trauma in remote Australia: a qualitative study of the Marurra-U family camp
Source: Lancet Reg Health West Pac. 2026 Sep 10;74:101974. doi: 10.1016/j.lanwpc.2026.101974 (PMC13584108; doi:10.1016/j.lanwpc.2026.101974)
Supplement: Supplementary file 3 [file mmc3.docx]

**Marurra-U Family Camp**

Feedback from parents

***Supplementary Figure 1. What is Broome Camp?*** *This figure and title were used to discuss analysis that led to theme 1 - The unique characteristics and design of the Marurra-U family camp – with parents and community members.*

***Supplementary figure 2. Parent Stories.*** *This figure and title were used to discuss analysis that led to theme 3 - Parents were motivated and empowered to support their children and communities to thrive – with parents and community members.*

***Supplementary figure 3. Kid Stories.*** *This figure and title were used to discuss analysis that led to theme 2 - Children actively engaged in camp activities, leading to improved individual, family and social wellbeing – with parents and community members.*

***Supplementary figure 4. How Broome Camp Ran.*** *This figure and title were used to discuss analysis that led to theme 4 - Two-way teams are effective in delivering child and family support – with parents and community members.*
